# Navigating data challenges in socioeconomic impact assessments of conservation regimes

**DOI:** 10.1111/cobi.14457

**Published:** 2025-04-01

**Authors:** Reem Hajjar, Johan A. Oldekop, Roberto Toto, Lucas Alencar, Samuel D. Bell, Katie Devenish, Duong T. Khuu, Mariana Hernandez‐Montilla, Suhyun Jung, Sandy Nofyanza, Lok Mani Sapkota

**Affiliations:** ^1^ Department of Forest Ecosystems and Society Oregon State University Corvallis Oregon USA; ^2^ Global Development Institute University of Manchester Manchester UK; ^3^ Department of Applied Economics Oregon State University Corvallis Oregon USA; ^4^ Department of Natural Resources and the Environment Cornell University Ithaca New York USA

**Keywords:** 30×30, data availability and accessibility, Global Biodiversity Framework, poverty and well‐being indicators, quasi‐experimental research designs, temporal and spatial resolution, 30×30, accesibilidad a datos, diseños de investigación cuasiexperimental, disponibilidad de datos, indicadores de bienestar, indicadores de pobreza, Marco Global de Biodiversidad, resolución espacial y temporal

## Abstract

Scholars are increasingly assessing the impact of conservation interventions at national and regional scales with robust causal inference methods designed to emulate randomized control trials (quasi‐experimental methods). Although spatial and temporal data to measure habitat loss and gain with remote sensing tools are increasingly available, data to measure spatially explicit poverty and human well‐being at a high resolution are far less available. Bridging this data gap is essential to assess the social outcomes of conservation actions at scale and improve understanding of socioenvironmental synergies and trade‐offs. We reviewed the kinds of socioeconomic data that are publicly available to measure the effects of conservation interventions on poverty and well‐being, including national census data, representative household surveys funded by international organizations, surveys collected for individual research programs, and high‐resolution gridded poverty and well‐being data sets. We considered 4 challenges in the use of these data sets: consistency and availability of indicators and metrics across regions and countries, availability of data at appropriate temporal and spatial resolutions, and technical considerations associated with data available in different formats. Potential workarounds to these challenges include analytical methods to help resolve data mismatches and the use of emerging data products.

## Abstract (es)

Retos para los datos de las evaluaciones de impacto socioeconómico en los regímenes de conservación

**Resumen:**

En la academia se evalúa cada vez más el impacto de las intervenciones de conservación a escala nacional y regional con métodos sólidos de inferencia causal diseñados para emular los ensayos de control aleatorios (métodos cuasiexperimentales). Aunque cada vez se dispone de más datos espaciales y temporales para medir la pérdida y ganancia de hábitats con herramientas de teledetección, se dispone de muchos menos datos para medir la pobreza y el bienestar humano espacialmente explícitos a alta resolución. Llenar este vacío de datos es esencial para evaluar los resultados sociales de las acciones de conservación a escala y mejorar la comprensión de las sinergias y compensaciones socioambientales. Revisamos los tipos de datos socioeconómicos que están disponibles públicamente para medir los efectos de las intervenciones de conservación sobre la pobreza y el bienestar, incluidos los datos de los censos nacionales, las encuestas representativas de hogares financiadas por organizaciones internacionales, las encuestas recopiladas para programas de investigación individuales y los conjuntos de datos de alta resolución sobre pobreza y bienestar. Consideramos cuatro retos en el uso de estos conjuntos de datos: la coherencia y disponibilidad de indicadores y métricas entre regiones y países, la disponibilidad de datos en resoluciones temporales y espaciales apropiadas, y las consideraciones técnicas asociadas con los datos disponibles en diferentes formatos. Entre las posibles soluciones a estos problemas se incluyen métodos analíticos que ayuden a resolver los desajustes de los datos y el uso de productos de datos emergentes.

## INTRODUCTION

Scholars increasingly call for rigorous impact evaluations of conservation interventions to determine their effectiveness (Baylis et al., 2016; Ferraro & Pattanayak, 2006). In response, various impact evaluation methods that compare conservation outcomes in treated units (where an intervention has taken place) with counterfactuals (comparable control sites with no intervention) have gained traction, and several guides for such evaluations have emerged. These guides include experimental and quasi‐experimental designs (Ferraro & Hanauer, 2014), statistical matching for conservation impact assessment (Ribas et al., 2021; Schleicher et al., 2020), and matching and panel regressions for assessing land‐cover changes (Jones & Lewis, 2015).

These techniques have been applied to assess the impacts of conservation interventions, including area‐based interventions (Alves‐Pinto et al., 2022), formalization of Indigenous land rights (Prioli & Walker, 2023), collective property (Baragwanath et al., 2023), and protected areas (Graham et al., 2021). These studies primarily focus on quantifying environmental impacts based on outcomes such as land‐cover change that can be readily measured using remote sensing data. In contrast, there have been relatively few large‐scale (national, regional, or global) analyses of conservation intervention impacts on human poverty and well‐being, despite their importance for understanding and informing just and sustainable conservation (Friedman et al., 2018; Löfqvist et al., 2023).

A key challenge to assessing the socioeconomic outcomes of conservation interventions is the availability and accessibility of socioeconomic data that are appropriate proxies for the phenomena of interest at temporal and spatial scales that correspond to the interventions in question. As conservation efforts ramp up in the wake of the new Global Biodiversity Framework, bridging existing socioeconomic data gaps is crucial for assessing social outcomes of conservation actions at scale (Choksi et al., 2023; Morgans et al., 2024). We sought to advance the conversation and improve the practice of such assessments by reviewing available and emerging data products and techniques for assessing socioeconomic outcomes of conservation interventions; discussing the uses, challenges, and limitations associated with these data; and highlighting ways for improving socioeconomic impact assessments.

## CHALLENGES AND OPPORTUNITIES OF EXISTING SOCIAL DATA

Although there have been calls for more experimental methods (e.g., randomized control trials [RCTs]) in conservation impact evaluation that replicate the rigor of studies of recent antipoverty interventions (Alpízar & Ferraro, 2020), results from RCTs can be overinterpreted or misunderstood (Deaton & Cartwright, 2018). Additionally, quasi‐experimental methods continue to be a more accessible approach for practical or ethical reasons (Baylis et al., 2016). Quasi‐experimental methods to evaluate conservation intervention impacts ideally require panel data (following the same units of observation across time before and after the intervention) in treatment and control areas. This design allows researchers to control for time‐invariant unobserved confounders, reducing differences between treated and control units to isolate the effect of the intervention. Equally, researchers need to be able to control for time‐variant confounders correlated with the intervention or outcome. Ideally, these data would be available at spatial resolutions matching the conservation intervention's potential impacts (e.g., at household or community levels).

Comprehensive socioeconomic panel data are largely unavailable at the spatial and temporal scales required to make inferences about the impacts of conservation interventions. Yet, many available socioeconomic data sets can be adapted to make causal inferences. These data sets fall into 4 categories (Table 1): national census data; representative household surveys funded by international organizations (e.g., the demographic and health survey [DHS] and the living standards and measurement survey [LSMS]); surveys collected for individual research programs (e.g., the International Forestry Resources and Institutions [IFRI], the Poverty and Environment Network [PEN] global data set); and emerging gridded high‐resolution human development and well‐being data sets. These data types have different limitations but also present substantial opportunities for assessing socioeconomic conservation impacts.

**TABLE 1:** A summary of different data types and their characteristics available for conducting socioeconomic impact assessments of conservation regimes.

Data type	Indicator availability and consistency	Temporal resolution	Spatial resolution	Format
Census	High	Medium (often every 5 or 10 years; good for panels)	High	Table format that needs to spatially link to administrative polygons based on unique identifiers
Nationally representative household surveys (e.g., DHS and LSMS)	Medium to high (some measures change over time)	Medium (high periodicity, but not panels)	Low	Point
Gridded	Limited availability (indicator or index choice); high consistency (across locations)	Low (for now)	High	Raster
Surveys published by research programs	Study dependent; low consistency	Usually low periodicity	High resolution, but very limited extent	Usually point

Abbreviations: DHS, demographic and health survey; LSMS, living standards and measurement survey.

Four factors should be considered when using these data sets. Three factors relate to study design (indicator availability and consistency and temporal and spatial resolution), and one relates to data formats. We also considered technical challenges posed by different data and used examples from our own work to show how they can potentially be overcome. As researchers consider how to link socioeconomic data to conservation interventions, data and methodological choices should be guided not only by what is available and feasible but also by the specific research questions and theories of change of the conservation intervention and what is knowable given these data.

### Indicator availability and consistency

Several socioeconomic variables are commonly used as proxies to construct poverty measures. However, compiling and analyzing household economic data can be complex and a barrier to examining conservation socioeconomic impacts. Consequently, many evaluations rely on ready‐made indexes to assess changes in household welfare, such as a wealth index (included in DHS) or Alkire and Foster's (2011) multidimensional poverty index (MPI). These data products have made research on poverty more accessible and, in the case of the MPI, reflect a recognition that poverty is multidimensional, encompassing not only economic deprivation but also health, education, housing, and other aspects (Sen, 1995). Often, poverty indexes are only available for recent decades. For example, in Cambodia, the DHS's wealth index is available from 2005 and the MPI is available from 2010, meaning researchers must calculate these metrics themselves for earlier DHSs. Although accompanying technical documents provide methodologies for estimating indexes, survey differences in earlier waves can affect the comparability of estimates over time.

An example of a ready‐to‐use index is the social lag index (*Índice de Rezago Social Longitudinal*) for Mexico. This index is used to consistently measure poverty in rural and urban localities over time (CONEVAL, 2023). It combines traditional poverty indicators (e.g., educational levels, access to electricity, sanitation) with access to internet, computers, and smartphones (CONEVAL, 2023). Sims and Alix‐Garcia (2017), for example, used a similar locality‐level data set to evaluate the impact of protected areas and payments for ecosystem services schemes on poverty alleviation and forest cover change. However, localities change over time, a challenge discussed below in “Spatial resolution.”

### Temporal resolution

Ideally, data are available at a periodicity that matches the conservation intervention being assessed: before the intervention for calculating baselines and preintervention trends and after the intervention at periods that allow for impacts to manifest. In the best scenarios, data are available in panel form, following the same units over time (although this presents its own methodological challenges [Brockington & Noe, 2021]). However, it is still possible to use nonpanel cross‐sectional data if data are available at multiple time points.

Census data can provide the required periodicity. Many nations undertake regular censuses that measure conservation‐relevant socioeconomic indicators, although nations differ in how they make census data accessible for research use (although data aggregated at higher administrative areas are often available freely online, high‐resolution data from some countries need to be licensed for research purposes at cost or obtained through visits to national statistics offices or connections to particular organizations). Similarly, representative household surveys (e.g., DHS data) are publicly available for many countries and often cover several periods. However, different households are typically sampled in each period, meaning these data are not panel data, but repeated cross‐sectional data. Therefore, it is not possible to track socioeconomic changes of individual households or sampling clusters over time. Yet, if the intervention is associated with administrative units that are consistent over time, one can construct panels if the data are representative at the level of that unit. Alternatively, it is possible to construct pseudo‐panels by grouping observations with exogeneous and time‐invariant variables that are available for all observations (Deaton, 1985; Verbeek, 2008). For example, Jung et al. (2019) used 4 cross‐sectional rounds of DHS data and household heads’ sex, birth year, and region as grouping variables to create cohorts so they could construct pseudo‐panel data to estimate household welfare impacts of forest concessions in Liberia.

Some freely available data only represent single periods. For example, IFRI data contain demographic, socioeconomic, and institutional information linked to forest management and governance for over 800 user groups in 18 countries (Ostrom et al., 2018). This data set has been used extensively to elucidate the relationships between forests and the communities that depend on them (e.g., Chhatre & Agrawal, 2009; Fischer et al., 2023; Persha et al., 2011), but the lack of repeated measurements strongly limits the strength of causal inferences. Nonetheless, cross‐sectional data can be used to attribute impacts to interventions if confounders are controlled carefully through regressions or matching or by using instrumental variable approaches (Souza‐Rodrigues, 2019). For example, Naidoo et al. (2018) used variation near protected areas and cross‐sectional DHS data to assess poverty impacts at a global scale.

Collecting new socioeconomic survey data specific to the intervention, question, and scale of interest across multiple periods is the most effective way to obtain optimal data for impact evaluations, but it is often logistically difficult, time consuming, and costly. For example, Campos‐Silva et al. (2021) surveyed 100 communities inside and outside protected areas over a 2000‐km^2^ area but did not have baseline data for comparison. Sometimes, baseline data have been collected but budgets cannot cover follow‐up surveys or a second round of surveys occurs too early to be able to detect socioeconomic changes. However, any available baseline data can be useful in designing survey data collection. Jung and Hajjar (2023) sampled the same households surveyed by the Ghanaian government in a separate baseline study to create panel data. They also utilized Ghana's LSMS surveys to identify control survey villages with similar biophysical and socioeconomic characteristics as the intervention villages to assess impacts of climate change mitigation programs on livelihoods.

### Spatial resolution

Spatial resolution and extent of available social data should match the hypothesized scale (in many cases, at a household, village, or district scale) and extent of intervention impacts. Census data, usually collected at the household scale and representative at all administrative levels, are ideal for measuring conservation impacts on local well‐being. However, in many countries, census microdata are difficult to access for privacy reasons and available data are often aggregated to higher administrative levels (e.g., province or region), making such data unsuitable for evaluating conservation impacts at local scales. However, exceptions do exist (e.g., Nepal and Brazil have published census data at very localized scales [Oldekop et al., 2019; den Braber et al., 2024]).

Publicly available household surveys, such as the DHS or LSMS, are designed to represent household information at broad administrative regions (e.g., provinces or countries). This creates a spatial mismatch between desired local‐scale analyses and available broad‐scale data. There are several context‐dependent approaches that practitioners may use to estimate local program effects when interventions and outcomes are spatially mismatched. There are 3 downscaling methods in which nationally representative, publicly available household survey data (e.g., DHS or LSMS) can be used to help resolve spatial mismatches, but these methods typically induce bias, information loss, or both.

In one method, it is possible to use a proximity buffer that associates treatment allocation with surveyed points within a radial proximity. A proximity buffer is used to look for DHS sample points within a particular distance of the treatment polygon's borders or centroid. The distance threshold is defined using some theory of change or prior knowledge on the likely spread of impacts or theory of change. The socioeconomic outcomes of those nearby points are then used to determine the program's local impacts on poverty. Although easily implemented, this method is highly sensitive to the stratified random sampling of the DHS, which is often sparse at fine scales. Pailler et al. (2015) used this approach, assigning all DHS clusters to community‐based natural resource management (CBNRM) treatments that were within 5 km of the managing‐village census tract. For anonymity purposes, DHS cluster points were randomly placed 5 km away from the sampling point, meaning any of the clusters within a 5‐km radius could have contained the true managing village. The authors argue that incidentally estimating treatment effects with non‐CBNRM villages as treated units “only bias[es] against finding a significant difference” between treated and untreated areas. However, estimates could be biased if there are large differences between non‐CBNRM villages inside and outside the 5‐km threshold. This approach should therefore be used for treatment specifications that are rich in data on confounders to show that untreated areas are socioeconomically similar across space.

A second approach is spatial interpolation, whereby a continuous surface of socioeconomic outcomes is estimated across a landscape based on the sampled point values and continuous data on variables that can be used to predict the outcome, such as population, elevation, and precipitation. However, interpolations in rural areas, where there are often fewer DHS points, can have lower precision. Predicted outcomes are only as good as the overlap between available predictors and the actual determinants of the outcome. For example, if the spatial prediction model only uses time‐invariant geospatial predictors, then the time variation of the predicted outcomes will only depend on the DHS points, which may be locally nonrepresentative. It is important to consider how predictors relate to the conservation program. To detect treatment effects, some spatial predictors must also be time‐variant mediators of the treatment. For example, consider a community forest program raises incomes of nearby households by creating local markets for nontimber forest products. If DHS sample villages weakly engage with the market and if the spatial predictors do not include proxies of local economic growth (like nightlights), then a treatment effect exists but is not detected by spatial interpolation. There are multiple tools to interpolate data (e.g., Gething et al., 2015; Wong et al., 2018), and novel machine learning methods have leveraged satellite imagery to downscale human development index (HDI) estimates to higher resolutions (discussed below) (Sherman et al., 2023).

The third approach is small area estimation (SAE), which is only possible when census data are available in addition to survey data. In an SAE, one uses statistical relationships between indicators that are common across a survey and census to predict a missing indicator of interest at the sample or census points (ADB, 2020; Corral et al., 2022). For example, if education and health can statistically predict income in a survey, education and health data from the census (with its continuous spatial coverage) can be used to impute missing information on income. Again, the predicted variable accuracy is only as strong as the statistical relationships on which the prediction is based. Incompatibility between the training and applied model may also arise if the primary sampling units (i.e., individuals vs. households) differ among data sets. Notably, SAE can be significantly more accurate than spatial interpolation because in SAE, actual local income determinants (e.g., household assets) are used. Thus, SAE adds crucial spatiotemporal variation in socioeconomic outcomes that reflect the impacts of the intervention.

These 3 methods can help produce data at the required spatial resolution for evaluating the socioeconomic impacts of conservation interventions. However, each involves biases and limitations, which can influence estimated treatment effects in ways that should be made transparent. When scale disparities between outcomes and intervention data are large, policy recommendations should be made with extreme caution.

An alternative to downscaling socioeconomic data is upscaling the treatment variable to match the available socioeconomic data. For example, researchers could aggregate treatment to the administrative units at which the socioeconomic data are representative and assign treatment by calculating the proportion of the unit that is treated (Jung et al., 2022). However, doing so can significantly reduce sample sizes if data are only representative at very high administrative levels. Importantly, impact estimates that use upscaling can fail to capture the spatial scale at which the program actually affects people.

An additional spatial challenge is that unit boundaries may change over time, making outcomes difficult to compare. For example, in 2017, Nepal Village Development Committees and municipalities were restructured to form local units (Bahl et al., 2022) without consistent identifiers to link previous and new local units. If original units cannot be tracked and assigned to their new formation, researchers cannot construct spatially explicit panel data. In this case, impact evaluations after 2017 in which existing population census data from 1991, 2001, 2011, and 2021 are used can only harmonize and compare these data at higher administrative levels.

New releases of high‐resolution, near‐global‐scale gridded poverty and well‐being data sets provide ready‐made downscaled data, enabling researchers to circumvent the above approaches. High‐resolution gridded data can be aggregated to whatever scale or unit of analysis of interest, providing researchers with substantial flexibility. Currently, there are 2 broad approaches to generating such data sets. The first approach harmonizes multiple global, national, and subnational socioeconomic data sets, including data on gross domestic product (GDP), HDI, and human population densities, and downscales these to a gridded format (e.g., CIESIN, 2022; Kummu et al., 2018). The most recent downscaled gridded data set is the Global Gridded Relative Deprivation Index at 1‐km resolution, available from the Center for Integrated Earth System Information (CIESIN).

The second approach involves the use of spatial interpolation and machine learning methods to predict poverty and well‐being at high resolution across large scales based on the modeled relationship between available socioeconomic data (e.g., from representative household surveys) and satellite imagery (see Burke et al. 2021) or other spatially continuous data sources (e.g., Chi et al. [2022] used Facebook connectivity as a proxy of wealth). This interpolation fills gaps in regions without survey or census data and generates wall‐to‐wall gridded poverty and well‐being estimates. Two recent multinational remotely sensed poverty and well‐being data sets are the microestimates of wealth for 132 low‐ and middle‐income countries (Chi et al., 2022) and global high‐resolution HDI estimates (Sherman et al., 2023) (available at 2.4‐ and 10‐km resolutions, respectively).

Despite the huge potential of these gridded data sets for evaluating social outcomes of conservation interventions, they have 2 key weaknesses. First, all current gridded data sets are available only for single periods (e.g., from 2010 to 2020 in the case of CIESIN's relative deprivation index) or a single year (e.g., Chi et al., 2022) and therefore cannot be used to measure changes over time. Although annualized high‐resolution HDI estimates (Sherman et al., 2023) are available from 2012 to 2021, these data cannot be used to compare HDI trends within country (e.g., comparing between treatment and control units) because all pixels within the same country exhibit the same trend. This is because the model was only trained using regional HDI estimates from 2019, and estimates for other years were backcast or forecast based on the national‐level change in HDI over time. Second, these data sets are models and therefore contain significant spatial uncertainties. Some contain models within models, whereby some predictors used in the main model are themselves estimates modeled from remotely sensed data. This can produce compounding errors that propagate into the final estimates but may not be transparent and consequently accounted for by the end user. For example, population densities from WorldPop (2024), which uses random forest models and characteristics from satellite imagery (e.g., land cover, nightlights, roads) to downscale administrative‐level population estimates from censuses, are a key component of the high‐resolution net migration estimates generated by Niva et al. (2023). Yet, modeled data are particularly difficult to validate in remote low‐population areas, which, although important for many conservation interventions, are often difficult to sample as part of household surveys and national censuses. These data must therefore be used with considerable caution (Pritchard et al., 2022).

The chosen scale of the spatial unit of analysis is of great consequence in conservation evaluation because a poorly chosen scale can significantly skew statistical results (Avelino et al., 2016). Researchers combining disparate spatial data sets should take care to aggregate data to a spatial unit that fits their research question, intervention, and data. Doing so means being aware of key scaling concepts from geographic science (ecological fallacy, modifiable areal unit problem, and spatial autocorrelation) and scale‐related methodological choices (unit of analysis, fixed effect scale, and clustered standard errors) that affect results. For background on how scaling impacts spatial information, see Jelinski and Wu (1996), Arbia (1986), and Fotheringham and Wong (1991). For more information on how the choice of spatial unit affects impact estimation, see Fletcher et al. (2023), Pietrzak (2014), Avelino et al. (2016), Garcia and Heilmayr (2022), Jones et al. (2018), and Blackman et al. (2024).

Researchers must also consider the hypothesized spatiotemporal extent of impacts, including indirect impacts and spillovers (positive or negative) affecting more distant localities. Where the theory of change identifies potential spillovers, researchers should aim to obtain data with sufficient spatial and temporal coverage to capture these spillovers and allow them to be incorporated into the analyses (e.g., D'Alberto et al., 2024). Lastly, a number of additional spatial econometric concerns surrounding spatial lags, standard errors of estimation, and nonclassical measurement error should also be considered (Abadie et al., 2023; Alix‐García & Millimet, 2023; Jain, 2020).

### Format

Census data, representative household surveys, and gridded data come in different formats, which can cause some challenges for data harmonization. Census data often come in different and multiple table formats (e.g., csv files) and often must be manipulated and merged with spatial boundary data for these administrative areas so that they can be linked to spatial data sets (e.g., forest cover data). This merging can usually be done with unique identifiers found in the census data and the spatial boundary data (e.g., municipality codes).

**FIGURE 1:**
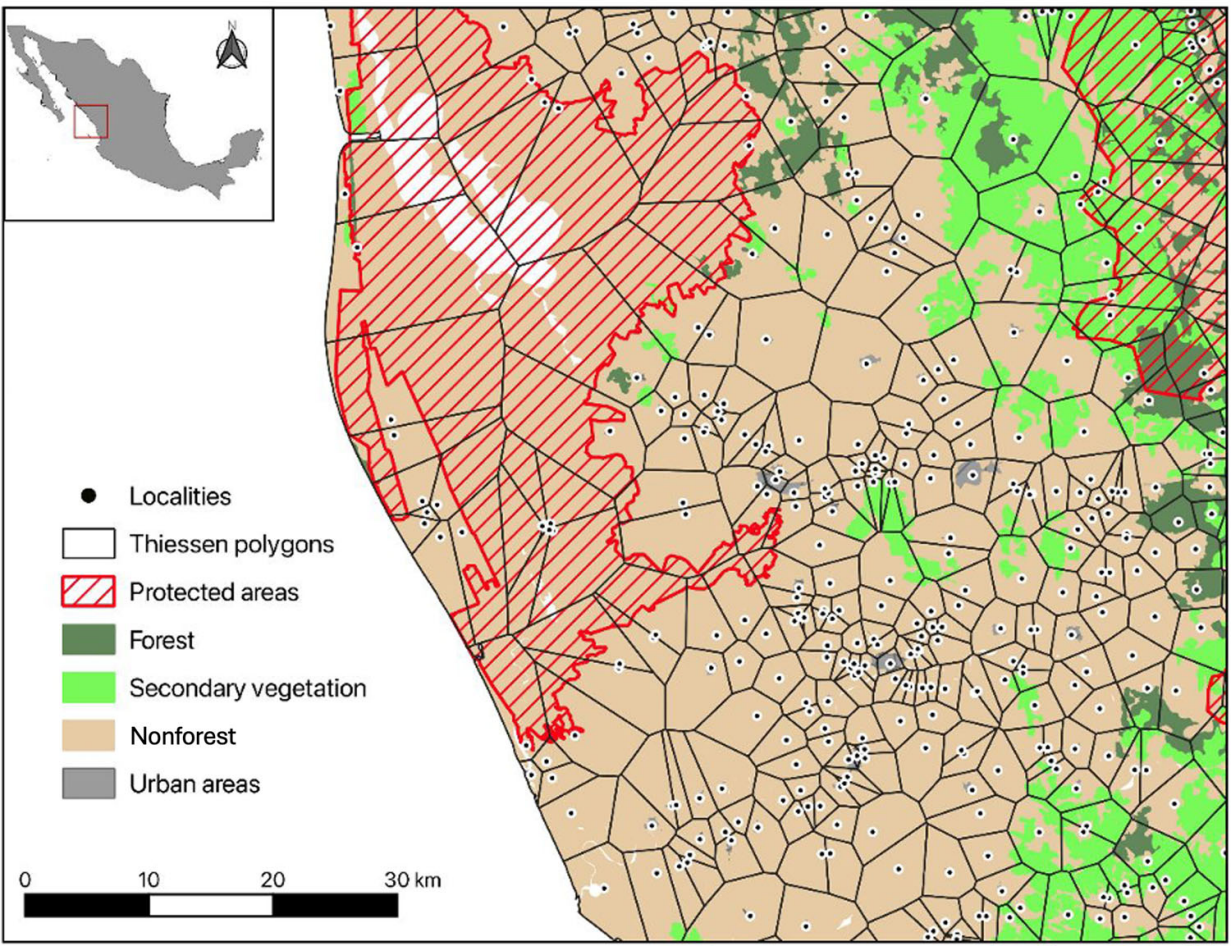
An example of Thiessen polygons (also known as Voronoi polygons) created from locality data in Mexico, which can be used to calculate land‐cover metrics.

Sometimes, spatial references are point data linked to individual localities rather than administrative boundaries (e.g., Mexico's social lag index). Census‐based data with point locations can be difficult to link with other spatial data (e.g., forest cover data) to create an area of influence. One way to address this issue is to create approximated areal units with Thiessen polygons (Figure 1). Thiessen polygons are built to create spatial boundaries when the data have no clear boundaries, such as point data. With these artificial boundaries, one can calculate land‐cover metrics, such as areas under forest recovery (secondary vegetation), area under environmental protection (e.g., protected areas), or any other spatialized conservation intervention. These boundaries are regarded as a good approximation of the real area under the influence of a given locality (or household or village) and have the additional advantages of avoiding overlaps between boundaries and creating a wall‐to‐wall mesh of polygons for the study area (Sims & Alix‐Garcia, 2017). The polygon mesh can change over time, as points appear and disappear in the data set. One approach to harmonizing the data set is to calculate area‐weighted averages with the first‐year mesh as baseline (e.g., Sims & Alix‐Garcia, 2017). In this way, one loses the spatial information (i.e., the new point location) but retains the statistical information (e.g., a poverty index).

Point data are commonly used to georeference representative household surveys and primary data collection efforts. However, because these surveys constitute a sample rather than a full census, it is not possible to use Thiessen polygons. Several ways to harmonize these data with other data exist, including demarcating buffers of a particular distance around individual points (e.g., Hall et al., 2022) or using other distance measures (see “Spatial resolution”).

### An illustrative example

**FIGURE 2:**
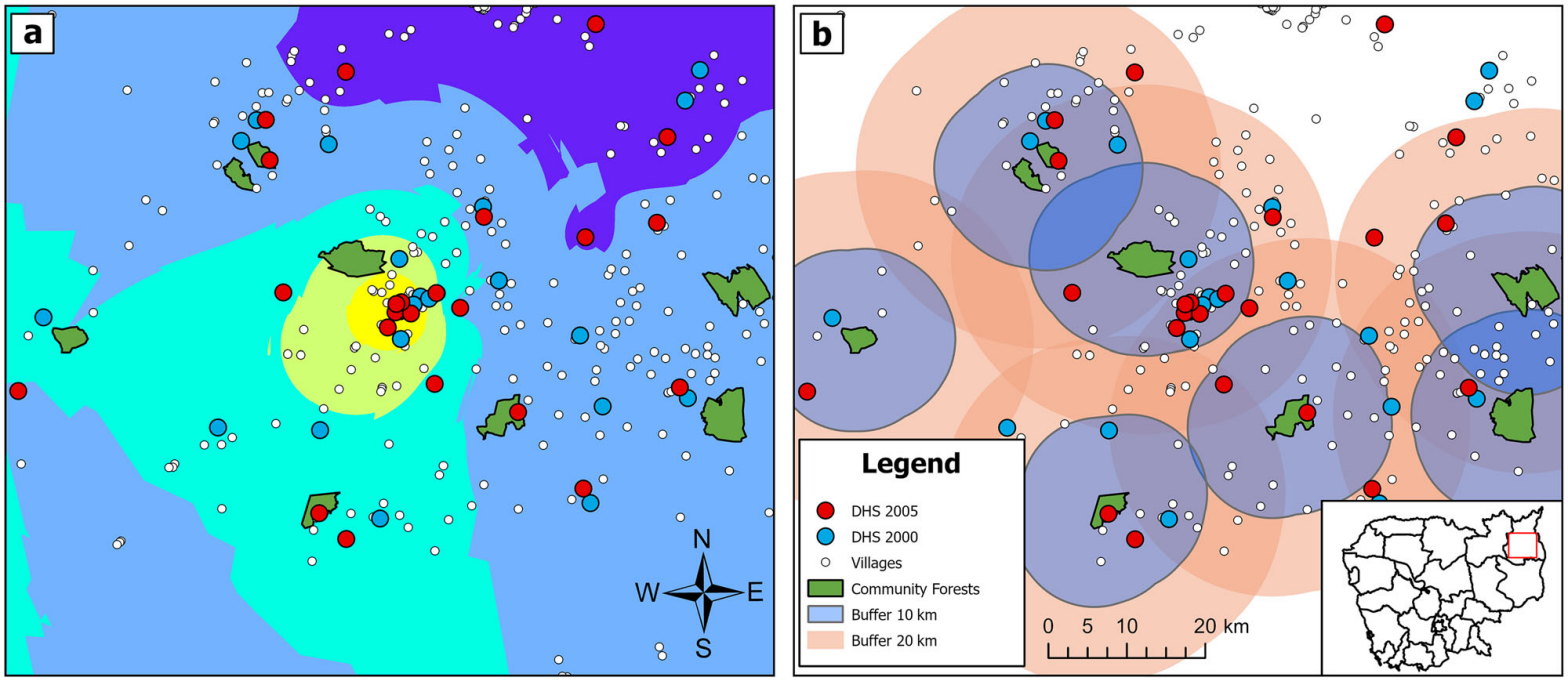
Fine‐scale spatial variation of demographic and health survey (DHS) cluster samples in Cambodia: (a) spatial variation among 2 DHSs, a census of villages and community forests at the subprovince scale, with kriging spatial interpolation of the 2005 DHS clusters’ wealth index as the background, and (b) capture of DHS points based on proximity buffers of 10 and 20 km to measure directly surveyed wealth outcomes over time (inset, location of clusters; red, northeast Ratanakiri province; black lines, province boundaries).

In Cambodia, if a researcher wants to measure the impact of community forests on socioeconomic outcomes in the villages managing those community forests (Figure 2), the outcomes must be tied precisely to each managing village. Ideally, the researcher would have census data over multiple periods that can be used to track poverty and well‐being at the village level to capture changes before and after the implementation of the community forest (and in selected controls). However, as in many countries, census data in Cambodia are only publicly available aggregated to higher administrative areas, not allowing for accurate village‐level estimations. The community forest program has been active in this area since the mid‐2000s, so more recent remote sensing gridded data would be less useful in isolating effects. The DHS data, however, are available from 2000, providing the necessary temporal resolution (although see “Indicator availability and consistency” on availability of indices over time).

Yet, the researcher faces 4 major challenges in using DHS data (Figure 2). First, assuming for simplicity that the managing village is the closest one to the forest, it is unlikely to be sampled in the DHS cluster since only a relatively small number of villages are sampled. Second, the DHS clusters are anonymized using a 5‐km random jitter, making the cluster's alignment with the true managing village nearly impossible because there are multiple villages within 5 km of most community forests. Third, the DHS clusters in different sampling years are independent random samples and distributed differently over space. As a result, the researcher is unlikely to observe a panel for any managing village even if true DHS locations are known. Finally, low variation in the rural observations of the wealth index and small sample sizes make interpolated wealth index values highly uncertain.

Of the 3 potential solutions reviewed in “Spatial resolution,” a proximity buffer is the best option in this context for several reasons. First, as shown in Figure 2a, a spatial interpolation of socioeconomic outcomes from the DHS has very low variation in rural areas, meaning it is not possible to estimate different effects of community forests on villages located in about half of Figure 2a (background dark blue). Second, census data are not available for 2 periods. So, although SAE is possible, it would not produce a panel data set and prevents estimation of effects over time. This leaves the proximity buffer solution as the next‐best feasible solution (Figure 2b), which fits the context well because it is known from interviews that community forests can benefit nearby villages regardless of whether a village is actively managing the forest. One can therefore compare socioeconomic outcomes between proximal and similar nonproximal DHS clusters to estimate a treatment effect of community forests. Additionally, one can test for sensitivity of proximity effects by varying buffer sizes. If it were not the case that community forests provide a general proximal benefit, one would move to a different solution, such as upscaling.

## CONCLUSION

Assessing the social impacts of conservation interventions is crucial for developing more equitable and sustainable conservation strategies. Although remote sensing data, such as those provided by Global Forest Watch, and conservation intervention data, such as the World Database on Protected Areas, are often freely accessible and enable large‐scale analyses of biophysical conservation outcomes, socioeconomic data at the needed scale of analysis are less accessible, standardized, and centralized. This disparity in data availability and quality hinders the ability to conduct comparable large‐scale analyses of conservation's social impacts.

However, there are ongoing efforts to improve socioeconomic data accessibility, standardization, and centralization. For example, IPUMS‐International's work aims to collect, preserve, document, harmonize, and distribute microdata globally for social science research. Their online platform enables users to select variables by country, sample, and year and extract customized and harmonized data. However, it is often challenging to use these data in evaluating conservation impacts, as most observations do not have spatially explicit information. National and international organizations involved in conservation (i.e., nongovernmental organizations, government agencies) should openly share spatial data related to conservation interventions (while following best practices for data protection and safeguarding privacy) and prioritize and invest in further socioeconomic data collection to enable independent evaluations. Conservation funders should ask for this.

Even when rigorous impact evaluations based on publicly available socioeconomic data are conducted, they may miss important nuances and dimensions of human well‐being. Many existing socioeconomic data sets do not capture gender dynamics, power relations, or other forms of inequality, which can significantly influence the distribution of conservation costs and benefits. Evaluations based on available data may also fail to fully capture the multidimensional nature of well‐being, including aspects such as social capital, cultural values, and subjective well‐being, which are crucial for understanding the full range of conservation impacts on local communities (Woodhouse et al., 2015). Although the socioeconomic data sets we discussed are useful for evaluations at subnational to national scales, the continued value of localized, context‐rich, and qualitative approaches to causal inference (Zavaleta Cheek et al., 2023) should not be underestimated.

We highlighted many limitations to acquiring and using existing data sets for social impact evaluations of conservation interventions. However, we also outlined several solutions, workarounds, and promising new products. Researchers can and should make use of such information while acknowledging inherent assumptions and uncertainties associated with different data sets. Increasing data availability means researchers can test the effect of data selection and chosen methods on results by repeating the analysis with different outcome variables (either different variables within the same data set or a different data set altogether) or using different methods or scales of analysis. In addition to data choices, researchers should pay close attention to study design. Constructing a theory of change or directed acyclic graphs (Ferraro & Hanauer, 2014) can help researchers better understand and identify potential confounders, appropriate treated and control units, and suspected causal relationships. Getting analyses wrong because of poor data quality or study design has ethical implications, particularly for the highly vulnerable populations often involved in or affected by conservation actions.

Much remains to be learned about how to make conservation interventions more socially effective, equitable, and just. By addressing the issues outlined above, we can develop a more comprehensive understanding of conservation outcomes and inform policy decisions that promote both environmental sustainability and human well‐being; data should not be the limiting factor to doing so.
